# Health Risk Assessment Indicators for the Left-Behind Elderly in Rural China: A Delphi Study

**DOI:** 10.3390/ijerph17010340

**Published:** 2020-01-03

**Authors:** Ruzhen Luo, Chunmei Zhang, Yanhui Liu

**Affiliations:** School of Nursing, Tianjin University of Traditional Chinese Medicine, Tianjin 301617, China; lrztjutcm@163.com (R.L.); suiyue801@163.com (C.Z.)

**Keywords:** left-behind elderly, health risk assessment, Delphi technique

## Abstract

In China, many young and middle-aged rural residents move to urban areas each year. The rural elderly are left behind. The number of the rural left-behind elderly is increasing with urbanization, but it is unclear which indicators can be used to assess their health condition. The health risk assessment index system was developed to improve the health level of the rural left-behind elderly. A two-round web-based Delphi process was used to organize the recommendations from fifteen Chinese experts in geriatrics, health management, social psychology who participated in this study. Meaningfulness, importance, modifiability, and comprehensive value of the health risk assessment indicators in the index system were evaluated. The effective recovery rates of the two-round Delphi were 86.67% and 92.31%, respectively. The judgement coefficient and the authority coefficient were 0.87 and 0.82, respectively. The expert familiarity was 0.76. Ultimately, the health risk assessment index system for the rural left-behind elderly consisted of five first-level indicators, thirteen second-level indicators, and sixty-six third-level indicators. The final indicators can be used to evaluate the health of the rural left-behind elderly and provide the basis for additional health risk interventions.

## 1. Introduction

Traditionally, Chinese elderly are taken care of by their children. But it becomes more and more difficult with the deepening of urbanization, domestic migration, and deconstruction of extended families. With the rapid development of the social economy, remarkable demographic transitions have taken place in China in the past three decades. Approximately 250 million rural residents (40% of the whole rural population) move to urban areas each year, most of them are young and middle-aged migrant workers. Therefore, a large number of rural children, women, and elderly are left behind [1,2]. Left-behind elderly refers to the elderly over 60 who live in rural areas while their children have been away from home six months at least [3]. According to the latest report of the National Bureau of Statistics, there are 127 million elderly people in rural China as of 2014. The number of left-behind elderly in rural areas has grown to 50 million, which account for 39.37% of the total rural elderly population. By 2020, the number of the elderly over 60 will increase to 255 million, and the number of elderly people living alone will increase to 118 million. The urbanization process and large-scale population migration will continue for a long time in China. By 2020, 300 million rural people at least will enter cities. Therefore, the number of the rural left-behind elderly will continue to increase.

The interaction between health and migration is complex, dynamic, and bidirectional. Migration not only affects the physical, mental, and emotional health and well-being of the migrants themselves, but also affects the health of the left-behind in the place of origin [4]. The elderly often suffer from chronic disease, such as heart disease, stroke, cancer, diabetes, arthritis, and falls [5]. Additionally, the challenges to mental health, such as depression, Alzheimer’s disease, and dementia, are the common health problems that the elderly often worry about [5]. Moreover, the elderly also face many social challenges besides physical and mental health problems [6].

The health condition of the left-behind elderly in rural areas is generally low in China, which is caused by low children support, high living pressure, low utilization rate of health resources, and incomplete low level of rural pension security in rural areas and so on. Chinese scholars generally believe that health is the most important problem to solve for the left-behind elderly in rural areas [7,8,9]. A survey of 1811 left-behind elderly in Thailand found that the health of the left-behind elderly in rural Thailand is poor. The results showed that the left-behind elderly in rural areas had a high risk of depression [10]. Antman surveyed 5247 Mexican left-behind elderly and 1483 Mexican non-left-behind elderly. The results showed that the risk of stroke and heart disease is higher in the left-behind elderly than that in the non-left-behind elderly. And the left-behind elderly were more likely to have negative emotions such as anxiety and depression than the non-left-behind elderly [11]. He et al. used the Geriatric Depression Scale to investigate the incidence of depression among 509 rural left-behind elderly in China, the results showed that the incidence of depression in the rural left-behind elderly was 36.49%. And the incidence of depression in women was 45.1%, which was higher than 33.43% in men [12]. Miltiades found that although the rural elderly obtained economic support from their children, the migration of children would directly affect the psychological status of the rural left-behind elderly; the elderly generally felt lonely and depressed [13].

Previous studies have also focused on the influencing factors of the health status among the left-behind elderly. A study of 1619 elderly people in Germany showed that age was the important influencing factor of mental health. The older the left-behind elderly, the greater the impact on their health [14]. Antman deemed that women, age, and the year of education were related to the physical and mental health of the left-behind elderly [11]. The survey was conducted among 6000 rural left-behind elderly over 60 in Gansu and showed that the prevalence rate of mental illness was 20.11%. Among all kinds of mental diseases, depression (9.20%), pain disorder (2.71%), and mood disorder due to physical condition (2.08%) rank the first three. The prevalence of female (242.89%) was significantly higher than that of male (119.55%), and the prevalence of unmarried (248.37%) was significantly higher than that of married (187.53%) [15]. Forlani et al. and Houtjes et al. pointed out that the gender, age, and the educational level were related to the physical and mental health of the elderly [16,17]. Williams et al. pointed out that religious beliefs, educational level, marital status, and family support can affect the quality of life of the elderly [18,19]. Xie et al. showed that types of chronic diseases, living style, and financial support of children of the left-behind elderly in rural areas are influencing factors of mental health [20]. A study among 509 rural left-behind elderly in China showed that the risks of depression were gender, visiting frequency of children, living environment, physical activity ability, types of chronic diseases, and the education level [12]. A survey among the elderly in Thailand found that, after controlling socio-demographic and economic variables, the elderly who had a migrant child were more likely to have poor mental health (OR = 1.10; 95% CI 1.05–1.17) than those children who had not migrated [21].

All in all, the prominent health problems not only reduce the quality of life of the rural left-behind elderly, but also increase the care pressure and the financial burden. The expenditure of the national long-term care for the elderly also increased. Present studies mainly focus on the physical and mental health status and its influencing factors of the rural left-behind elderly. There are few studies exploring the health risk assessment indicators for the rural left-behind elderly.

Health ecology model is a theoretical model originated from ecology. It is an important model to solve human health problems in preventive medicine and public health [22]. The model shows that people’s health is influenced by individual factors, social environment, medical and health services. The health ecology model includes five layers: the core layer is the personal traits, the second layer is the behavior characteristics, the third layer is the interpersonal network, the fourth layer is the living and working conditions, and the fifth layer is the social, economic, cultural, and related policies. At present, it has been applied to the studies of obesity, nutrition, smoking cessation control, self-management of diabetes, and the explanations of healthy influencing factors [23,24,25,26]. In the analysis of influencing factors, the health ecology model not only involves individual physiology, psychology, life style, physical environment, and social environment, but also emphasizes the interaction of these factors. Therefore, the purpose of this study is to develop the health risk assessment index system for the left-behind elderly dependant on the health ecology model so that the health problems can be found earlier and the ability of assessing risk factors of elderly diseases can be improved. Thus, the health condition and quality of life of the left-behind elderly will be improved. Healthy ageing will be achieved easily.

## 2. Materials and Methods

The Delphi method was used, which was developed by Dalkey and Helmer in the 1950s [27]. It is an acknowledged method to gather consensus of opinion and choice about a topic [28]. The Delphi method is a structured iterative process that uses repetitive administration of questionnaires to gather information [29]. The Delphi method is widely used to develop assessment indicators in healthcare and achieve convergence opinions among experts and participants on specific topics [29,30]. The consensus process incorporated a two-round web-based Delphi method, which took place between July 2018 and November 2018.

### 2.1. Experts Selection and Delphi Implemention

Experts were selected to reflect the components of the health risk assessment. It is composed of geriatricians, health management experts, and social psychologists. There are no guidelines for the sample size of the Delphi study [31]. However, in general, the more panelists participate, the more reliable the group judgment will be [32]. Therefore, fifteen experts from six provinces and cities in China were selected. There was diverse geographical representation among the panel members, which came from various provinces of China including Beijing, Tianjin, Shanxi, Fujian, Zhejiang, and Jilin. The selection criteria were as follows: (1) working for 10 years and over in geriatrics, health management, social psychology, and other works, with rich professional theory and practical experience; (2) deputy senior and above technical titles; (3) informed consent and voluntary participation in this study. A key component of the Delphi technique is the anonymity of the expert panel members. Thus, none knew the identity of other panel members. 

The informed consent for inclusion were given to all subjects before they participated in the study. The study was conducted in accordance with the Declaration of Helsinki, and the protocol was approved by the medical ethics committee of Tianjin University of Traditional Chinese Medicine.

The initial questionnaire with candidate indicators was pre-tested with three physicians (who were not recruited to the Delphi panel) to anticipate the average completion time and for clarity.

The Delphi questionnaires were sent out by e-mail. After summarizing and analyzing the experts’ opinions in Round I, the addition, deletion, and modification of the previous round of questionnaire items were completed through a literature review and group discussion. The second round of expert correspondence was carried out. When the experts’ opinions tended to be consistent, the correspondence ended.

### 2.2. Questionnaire Preparation

Based on the health ecology model, the five first-level indicators of the health risk assessment index system for the rural left-behind elderly were: personal characteristics, behavior characteristics, interpersonal network, living and working conditions, social, economic, cultural, and related policies. According to the definition of the first-level indicators, thirteen second-level indicators were determined.

Bibliography retrieval was conducted. “Old people, elderly, left behind, countryside, health, physical health, mental health, social adjustment” were used to search literature in English databases such as Web of Science, Pubmed, EMBASE, CINAHL and Chinese databases such as Wanfang Database, CNKI, and Weipu Database. On the basis of the literature research, health risk assessment indicators for the rural left-behind elderly were extracted. From this, an expert inquiry paper was compiled. 

**Figure 1:**
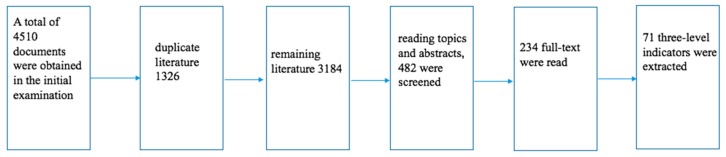
Steps taken to implement a bibliography retrieval.

Through the retrieval of literature 4510, deletion of duplicate literature 1326, remaining literature 3184, reading topics and abstracts, 482 were screened, 234 full-text were read, 71 three-level indicators were extracted, which are shown in Figure 1.

A list of 71 indicators was included in the first-round questionnaire. These indicators were divided into personal traits, behavioral characteristics, interpersonal network, living conditions, social, economic, cultural, and related policies, which describe the health risks of the rural left-behind elderly. The first round of the expert inquiry included four parts: (1) the introduction of the subject, purpose, significance, and instructions of this Delphi; (2) basic information of experts, including age, position, title, length of service, work, and professional field, etc.; (3) expert opinion of the first-level, second-level, and third-level indicators of the health risk assessment for the rural left-behind elderly. Each indicator was accompanied by a column of importance judgment and revision comments for experts to put forward suggestions of the indicators. The Likert 5-level scoring method was adopted: “very important”, “important”, “general important”, “unimportant”, and “very unimportant” were assigned 5, 4, 3, 2, and 1 points, respectively. (4) Expert familiarity and judgment are used to understand the authority of the experts. Experts familiarity can be divided into five grades: very familiar, relatively familiar, generally familiar, a little familiar, and unfamiliar and assigned 0.9, 0.7, 0.5, 0.3, and 0.1, respectively. Expert judgment criteria are shown in Table 1. 

The indicators were revised according to the experts’ opinions. In the second round of expert inquiry, experts were invited to re-comment the newly revised indicators. All experts who had participated in Round I were sent an email with the second-round questionnaires. But if the expert chose “know a little” and “not familiar” with the research content in the first round of the inquiry, the expert was not invited in the second round. The consensus of indicators in Round I, the modified and new indicators suggested by the experts in Round I were included in Round II [33]. The experts were asked to re-score each indicator using the same criteria based on their own opinion.

### 2.3. Statistical Analysis

The data were input by Excel (Microsoft Corporation, Redmond, WA, USA) and analyzed by SPSS17.0 (IBM Cor- poration, Chicago, IL, USA). The general information of experts is expressed by frequency and percentage; the measurement data are expressed by mean and standard deviation; the enthusiasm of experts is expressed by the rate of valid questionnaires (the number of returned questionnaires/the number of total questionnaires*100%); and the coefficient of experts’ authority (Cr) is determined by the coefficient of experts’ judgement (Ca) and experts’ familiarity (Cs). The degree of concentration of experts’ opinions is expressed by importance assignment mean (significance) and standard deviation (SD). The importance assignment mean significance <3.5 is taken as the criterion for deleting indicators. The coordination degree of experts’ opinions is expressed by the coefficient of variation (CV) and the coordination coefficient (W). CV >0.25 is the criterion for deletion [34]. Yaahp software was used to calculate the weight of each index in the health risk evaluation index system of the rural left-behind elderly [35].

## 3. Results

### 3.1. The Authority of Experts

The characteristics of the experts who participated in the study are shown in Table 2. Fifteen experts were invited to participate in the Delphi study, thirteen (86.67%) experts gave feedback in Round I, twelve (92.31%) out of thirteen experts accepted the invitation and gave feedback in Round II.

### 3.2. The Authority of Experts

The judgment criteria and familiarity of experts in the two-round Delphi study are shown in Table 3 and Table 4. The judgment coefficient of experts is 0.87. The expert familiarity is 0.76. The authority coefficient of experts is 0.82.

### 3.3. Delphi Round I

In Delphi Round I, there are five first-level indicators including personal traits, behavioral characteristics, interpersonal network, living conditions, social, economic, cultural, and related policies. There are thirteen second-level indicators which includes native traits, disease susceptibility, psychosocial characteristics, habits, health behavior, family interpersonal network, community interpersonal network, social interpersonal network, conditions for medical treatment, socio-economic status, political environment, economic environment, and cultural environment. Seventy-one third-level health risk assessment indicators for the rural left-behind elderly were formed. The indicators reached consensus in Round I were included in the questionnaire of the Round II. 

Combined with the statistical results and the suggestions of the experts in Delphi Round I, six three-level indicators were deleted, such as “the length of telephone conversation with children, the gender of children, the intensity of labor services”, and eight three-level indicators were added, such as “children bear medical expenses”, “social assistance”, “cultural and recreational activities”. At the same time, fifteen third-level indicators were modified, such as “daily living ability” was changed to “activities of daily living” and “alcoholism” was changed to “drinking”. 

### 3.4. Delphi Round II

**Figure 2:**
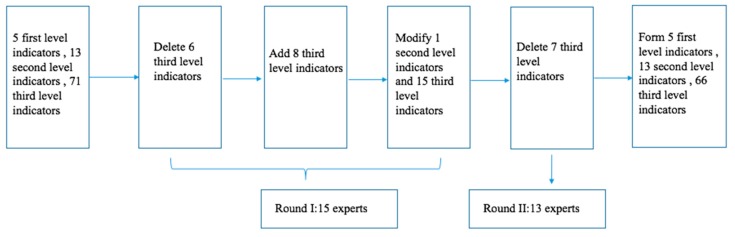
A Delphi study on the health risk assessment index system for the left-behind elderly.

After Delphi Round I, there are five first-level indicators, thirteen second-level indicators, and seventy-three third-level indicators in the health risk assessment index system. In Delphi Round II, experts have a high concentration on indicators at all levels, indicating that experts tend to agree. So, the correspondence ends. Combined with the expert opinions in Round II, after the literature review and group discussion, seven three-level indicators, such as “number of children”, “frequency of communication with children”, “family members as medical workers”, were deleted. Finally, the health risk assessment index system for the rural left behind elderly was formed, which includes five first-level indexes, thirteen second-level indexes, and sixty-six third-level indexes. Two rounds of Delphi process are shown in Figure 2. Also, in Delphi Round II, the weight of indicators were given by experts.

### 3.5. Final Indicators

After the two-round Delphi study, five first-level indicators, thirteen second-level indicators, and sixty-six third-level indicators were compiled. The results are presented in Table 5. In the first-level indicators, personal traits (0.248) and behavioral traits (0.248) were the most important indicators. In the second-level indicators, the largest weight value is the medical condition (0.149). The largest weight value of the three-level indicators is the assistance provided by medical institutions (0.111).

## 4. Discussion

Guided by the health ecology model, the health risk assessment index system for the rural left-behind elderly was developed. The weights of the indicators indicate their importance [36]. Personal traits and behavioral traits were the most important first-level indicators in this study. At present, relevant studies have also confirmed that personal traits have impact on the health of the left-behind elderly. Glaesmer et al. surveyed 1659 elderly people in Germany, the results showed that age affects their mental health. The age of stay-at-home and education were related to their lower physiological and mental health level [14]. Regarding the behavioral characteristics, He et al. showed that the depression of 509 rural left-behind elderly was related to their physical activity ability [12]. Through the analysis of 209 left-behind elderly in Henan Province, Zhao and Zhang found that the quality of life of the rural left-behind elderly is affected by many factors, including age, negative life events, per capita annual income, social support, marriage, and family [37]. The second-level indicator with the largest weight value is the medical condition. Income level can affect the health status of the elderly. The elderly with different income have different medical behavior. The higher the income and the medical condition, the better their health condition [38]. As some researchers point out, children’s migration contributes to the material well-being of their parents and better economic status is associated with less adverse health outcomes [7,39]. Some studies also show that heavy living burden and shortage of available medical service resources lead to higher incidence of stroke and heart disease for the left behind elderly. Also, when negative emotions such as loneliness increase, the mental health and self-rated health status reduce [21,39]. The three-level indicator with the largest weight value is the assistance provided by medical institutions. It indicates that the left-behind elderly trust the professional guidance given by medical institutions. So medical institutions, especially medical staff, should provide more medical and nursing services for the elderly. There is a significant positive correlation between the level of rural medical service and the objective health status of the rural left-behind elderly [40].

Valid and reliable measures depend on high-quality data [41]. This study used a series of scientific measures. But the Delphi method has its limitations, including purposeful selection of the panelists, attrition rate, and non-response bias although the two-round Delphi study and reminder letters helped to prevent attrition. A separate study is required to determine minimum data for implementing the measures [42]. Secondly, there were no left-behind elderly participating in the development process. The left-behind elderly with health problems should be invited in the development process in future research. Moreover, the study was anonymous, so we were unable to find changes between two rounds within respondents. Finally, the study investigators assumed that all the Delphi experts are familiar with the relevant knowledge of the research theme. However, it is conceivable that not all the experts were aware of the measures that reached agreement.

## 5. Conclusions

The Delphi technique has been used to develop the health evaluating indicators in previous studies. On the basis of literature research and expert inquiries, a health risk assessment index system for rural left-behind elderly was formed with five first-level indicators, thirteen second-level indicators, and sixty-six third-level indicators. The recommending indicators of this study are intended to provide a comprehensive tool to evaluate the health risk of the rural left-behind elderly. It also can be used for medical workers and health managers to identify the health risks of the rural left-behind elderly earlier. In particular, policy-makers can design future care systems for rural older adults. Therefore, the quality of life and health level of the rural left-behind elderly can be improved, the healthy ageing of the rural left-behind elderly can be promoted.

## Figures and Tables

**Table 1:** Expert judgment criteria.

Judgment Basis	Degree of Influence on Expert Judgment		
	Big	Medium	Small
theoretical analysis	0.1	0.1	0.1
practical experience	0.5	0.4	0.3
peer understanding	0.3	0.2	0.1
intuitive perception	0.1	0.1	0.1

**Table 2:** Main characteristics of the expert in two rounds of the Delphi study.

Characteristics	Experts in Round I (*n* = 13)	Experts in Round II (*n* = 12)
**Age**	*M* = 46.92, *SD* = 5.63	*M* = 47.67, *SD* = 5.18
**Gender**		
Male	2 (15.38%)	2 (16.67%)
Female	11 (84.61)	10 (83.33%)
**Province**		
Beijing	1 (7.69%)	1 (8.33%)
Tianjin	5 (38.46%)	4 (33.33%)
Shanxi	1 (7.69%)	1 (8.33%)
Fujian	3 (23.08%)	3 (25%)
Zhejiang	2 (15.38%)	2 (16.67%)
Jilin	1 (7.69%)	1 (8.33%)
**Speciality**		
Geriatrics	7 (53.85%)	6 (50%)
Health management	4 (30.77%)	4 (33.33%)
Social psychology	2 (15.38%)	2 (16.67%)
**Professional title**		
Senior professional title	5 (38.46%)	5 (41.27%)
Sub-senior professional title	8 (61.54%)	7 (58.33%)

**Table 3:** Expert judgment criteria in Round I.

Judgment Basis	Big		Medium		Small	
	Number	Frequency	Number	Frequency	Number	Frequency
Theoretical analysis	11	84.62%	2	15.38%	0	0.00%
Practical experience	8	61.54%	5	38.46%	0	0.00%
Peer understanding	3	23.08%	8	61.54%	2	15.38%
Intuitive perception	0	0.00%	6	46.15%	7	53.85%

**Table 4:** Expert familiarity in Round I and Round II.

Expert Familiarity	Very Familiar	Relatively Familiar	Generally Familiar	A Little Familiar	Unfamiliar
Round I (Number of experts)	6	5	2	0	0
Round II (Number of experts)	6	5	1	0	0

**Table 5:** Indicators of the health risk assessment index system for the rural left-behind elderly.

First-Grade Index (Weight)	Second-Grade Index (Weight)	Third-Grade Index	*M* ± *SD*	*CV*	*Weight*
Personal traits (0.248)	Native traits (0.124)	Gender	4.33 ± 0.65	0.15	0.062
		Age	5	0	0.062
	Disease susceptibility (0.124)	Inheritance factor	4.75 ± 0.45	0.10	0.015
		Nutritional status	4.75 ± 0.45	0.10	0.015
		Suffering from chronic diseases	4.83 ± 0.39	0.08	0.026
		Types of chronic diseases	4.83 ± 0.39	0.08	0.026
		Severity of chronic diseases	5	0	0.041
Behavioral characteristics (0.248)	Psychosocial characteristics (0.062)	Character	4.50 ± 0.67	0.15	0.009
		Coping style	4.75 ± 0.45	0.10	0.017
		Hobbies and interests	4.08 ± 0.90	0.22	0.004
		Negative life events	4.92 ± 0.29	0.06	0.027
		Ageing attitudes	4.33 ± 0.89	0.21	0.006
	Habits (0.062)	Eating habits	4.83 ± 0.39	0.08	0.028
		Smoking	4.92 ± 0.29	0.06	0.028
		Drinking	5	0	0.053
		Sleep condition	4.67 ± 0.49	0.11	0.015
	Health behavior (0.124)	Medication compliance	5	0	0.031
		Health knowledge	4.75 ± 0.45	0.10	0.009
		Physical exercise	4.92 ± 0.29	0.06	0.022
		Active medical seeking behavior	4.83 ± 0.39	0.08	0.012
		Activities of daily life	4.75 ± 0.45	0.10	0.016
Interpersonal network (0.150)	Family Interpersonal Network (0.090)	Marital status	4.58 ± 0.52	0.11	0.003
		Spouse health	4.75 ± 0.45	0.10	0.004
		Family relationship	4.83 ± 0.39	0.08	0.006
		Family size	3.92 ± 0.90	0.23	0.001
		Living style	4.50 ± 0.67	0.15	0.002
		Look after by spouse	4.75 ± 0.45	0.10	0.004
		Frequency of children returning home	3.92 ± 0.67	0.17	0.001
		Physical condition of children	4.08 ± 0.90	0.22	0.001
		Economic status of children	4.50 ± 0.52	0.12	0.003
		Number of outgoing children	3.75 ± 0.75	0.20	0.001
		Take care of grandchildren	4.17 ± 0.84	0.20	0.001
		Number of grandchildren to take care	3.92 ± 0.67	0.17	0.001
		Years of left behind	4.33 ± 0.78	0.18	0.002
	Community interpersonal network (0.030)	Frequency of communication with neighborhood	4.17 ± 0.58	0.14	0.004
		Neighborhood friendship	4.50 ± 0.67	0.15	0.008
		Assistant for neighborhood	4.33 ± 0.78	0.18	0.008
		Frequency of communication with relatives	4.08 ± 0.67	0.16	0.002
		relationship	4.42 ± 0.79	0.18	0.005
		Relatives’ help	4.17 ± 0.84	0.20	0.004
	Social Interpersonal Network (0.030)	Assistance provided by medical institutions	4.83 ± 0.58	0.12	0.111
		Access to external information	4.42 ± 0.67	0.15	0.037
Living conditions (0.223)	Conditions for medical treatment (0.149)	Sources of medical expenses	4.67 ± 0.49	0.11	0.007
		Children bear medical expenses	4.08 ± 0.67	0.16	0.002
		Utilization of health resources	5	0	0.016
		Traffic time of go to doctor	5	0	0.016
		Regular physical examination	5	0	0.016
		Technical level of medical staff	4.33 ± 0.49	0.11	0.004
		Service attitudes of medical staff	4.17 ± 0.84	0.20	0.003
		Infrastructure health facilities	4.83 ± 0.58	0.12	0.010
	Socio-economic status (0.074)	Degree of education	4.67 ± 0.49	0.11	0.007
		Labor intensity	4.25 ± 0.75	0.18	0.011
		Residential environment	4.92 ± 0.29	0.06	0.001
		Engage in sideline work	3.42 ± 0.52	0.15	0.017
		Economic source	5	0	0.011
		Family economic situation	4.83 ± 0.39	0.08	0.005
		Social assistance	4.42 ± 0.79	0.18	0.005
		New rural social pension insurance	4.50 ± 0.52	0.12	0.028
Social, economic, cultural, and related policies (0.131)	Political environment (0.056)	Social security policy	5	0	0.028
		Public health policy	5	0	0.019
	Economic environment (0.056)	Local economic development level	4.58 ± 0.52	0.11	0.037
		Local economic burden of medical care	4.75 ± 0.45	0.10	0.002
		Cultural and recreational activities	3.67 ± 0.78	0.21	0.003
	Cultural environment (0.019)	Nationality	3.92 ± 0.79	0.20	0.007
		Ideology	4.33 ± 0.99	0.23	0.001
		Hygiene concept	3.42 ± 0.67	0.20	0.007
		Religious belief	4.33 ± 0.49	0.11	0.062
